# SARS-CoV-2 Infection and Disease Modelling Using Stem Cell Technology and Organoids

**DOI:** 10.3390/ijms22052356

**Published:** 2021-02-26

**Authors:** Marta Trevisan, Silvia Riccetti, Alessandro Sinigaglia, Luisa Barzon

**Affiliations:** Department of Molecular Medicine, University of Padova, 35121 Padova, Italy; marta.trevisan@unipd.it (M.T.); silvia.riccetti@unipd.it (S.R.); alessandro.sinigaglia@unipd.it (A.S.)

**Keywords:** lung, airway epithelial cells, central nervous system, gastrointestinal tract, cardiovascular system, SARS-CoV-2, ACE2, tropism, innate immune response, pathogenesis

## Abstract

In this Review, we briefly describe the basic virology and pathogenesis of SARS-CoV-2, highlighting how stem cell technology and organoids can contribute to the understanding of SARS-CoV-2 cell tropisms and the mechanism of disease in the human host, supporting and clarifying findings from clinical studies in infected individuals. We summarize here the results of studies, which used these technologies to investigate SARS-CoV-2 pathogenesis in different organs. Studies with in vitro models of lung epithelia showed that alveolar epithelial type II cells, but not differentiated lung alveolar epithelial type I cells, are key targets of SARS-CoV-2, which triggers cell apoptosis and inflammation, while impairing surfactant production. Experiments with human small intestinal organoids and colonic organoids showed that the gastrointestinal tract is another relevant target for SARS-CoV-2. The virus can infect and replicate in enterocytes and cholangiocytes, inducing cell damage and inflammation. Direct viral damage was also demonstrated in in vitro models of human cardiomyocytes and choroid plexus epithelial cells. At variance, endothelial cells and neurons are poorly susceptible to viral infection, thus supporting the hypothesis that neurological symptoms and vascular damage result from the indirect effects of systemic inflammatory and immunological hyper-responses to SARS-CoV-2 infection.

## 1. Introduction

Severe acute respiratory syndrome coronavirus 2 (SARS-CoV-2) emerged in December 2019 in Wuhan, China, where clusters of cases of severe interstitial pneumonia were identified [1,2,3,4,5,6]. The virus has rapidly spread worldwide leading the World Health Organization (WHO) to declare COVID-19 a pandemic threat in March 2020. One year after 31 December 2019, when the first infections were identified, 191 countries have reported COVID-19 cases, accounting for over 82 million cases and 1.8 million deaths worldwide [7]. 

**Figure 1:**
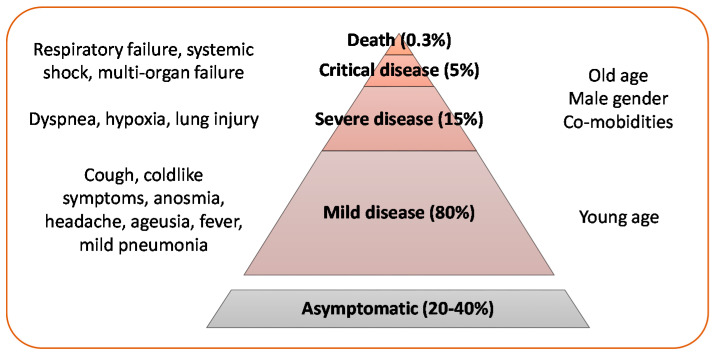
Clinical presentation of SARS-CoV-2 infection: About 20–40% of SARS-CoV-2 infections are asymptomatic, especially in young individuals [10,11,12,13]. Mild symptoms are reported in about 80% of symptomatic infections (with or without mild pneumonia), severe disease in about 15%, critical disease in 5% [14]. The risk of death from SARS-CoV-2 infection has been estimated as 0.3% [15]. Elderly age, male gender, and presence of co-morbidities are risk factors for severe disease [16,17].

Like the other respiratory coronaviruses, SARS-CoV-2 is mainly transmitted by respiratory droplets, while the role of airborne and fecal–oral transmission and direct contact with contaminated surfaces is uncertain [8,9]. Clinical presentation of the disease caused by SARS-CoV-2, named coronavirus disease 2019 (COVID-19) by WHO, ranges from a mild disease to a potentially fatal acute respiratory distress syndrome (Figure 1). 

In addition, SARS-CoV-2 infection has been associated with a variety of clinical conditions, including a new multisystem inflammatory syndrome in older children, manifested by severe abdominal pain, cardiac dysfunction and shock [18], pulmonary fibrosis, neurological and neuropsychiatric complications (cerebrovascular events, encephalitis, Guillain Barré syndrome, altered mental status) [19], acute pancreatitis, coagulopathy, renal failure, diabetes, and dermatological lesions [20,21]. 

Unprecedented research efforts have rapidly allowed the understanding of the epidemiology, biology, and pathogenesis of SARS-CoV-2 infection and the development or antiviral therapies and prophylactic vaccines [22,23,24]. Generation of experimental models of SARS-CoV-2 infection and disease has been crucial to understand the biology of the virus, to dissect the pathogenic mechanisms of disease, and to develop antiviral drugs. While animal models are still required in preclinical studies for vaccine development [25], in vitro models established from stem cells and organoids are increasingly being used as cruelty-free and valuable systems for pathogenesis and drug discovery studies [26] and provided a significant contribution to the understanding of SARS-CoV-2 infection and COVID-19 pathogenesis [27]. In this review article on the use of stem cell technology and organoids for COVID-19 modelling and drug discovery, we highlight the contributions and the potentialities of these biotechnological tools to the advancement of knowledge on disease pathogenesis. In addition, we contribute a brief update on the genetic and biological features of SARS-CoV-2 and on COVID-19 pathogenesis.

## 2. Genetic and Biological Features of SARS-CoV-2

Coronaviruses are enveloped, single-stranded, positive-sense RNA viruses that infect a variety of vertebrate host species. Like the two other highly pathogenic human coronaviruses SARS-CoV and MERS-CoV, SARS-CoV-2 is classified in the *Betacoronavirus* genus of the *Coronaviridae* family, and, together with SARS-CoV, it belongs to the *Severe acute respiratory syndrome-related coronavirus* species [28]. The virus shares 79.6% sequence identity with SARS-CoV, which was responsible for outbreaks of severe acute respiratory syndrome (SARS) in Guangdong Province, China, 2002–2003 [6], and 96% identity with other SARS-like betacoronaviruses of bat origin from China [2,3]. Phylogenetic and phylodynamic analyses of full SARS-CoV-2 genomes showed the emergence of several evolutionary lineages, which allowed to track the worldwide dispersal and evolution of the virus [29,30].

**Figure 2:**
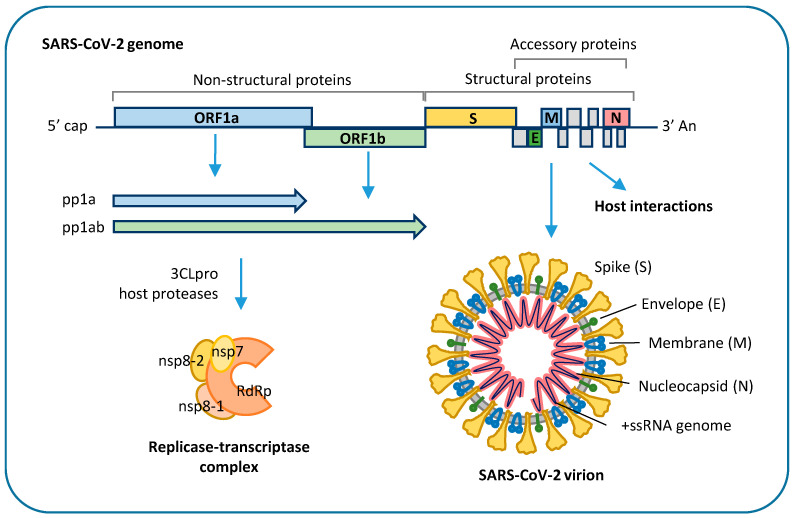
Schematic representation of SARS-CoV-2 genome and viral proteins: The 5′-terminal part of SARS-CoV-2 genome encodes polyproteins pp1a and pp1ab, which are cleaved into 16 different nonstructural proteins (nsp), which generate the replicase-transcriptase complex. Structural proteins and accessory proteins are encoded in the 3′-terminal part of the genome. Structural proteins, i.e., spike (S), envelope (E), membrane (M), and nucleocapsid (N), are assembled with a 30 kb positive-sense, single-stranded RNA (+ssRNA) genome to generate enveloped SARS-CoV-2 virions. Accessory proteins are thought to be involved in host antiviral response modulation and viral pathogenesis. ORF: open reading frame; 3CLpro: 3-chymotrypsin-like protease; RdRp: viral RNA-dependent RNA polymerase.

SARS-CoV-2 genome is about 30 kb in size, has a 5′-cap and a 3′-poly(A) tail, which allow immediate translation by the host cell, and 5′- and 3′-untranslated regions, which are involved in the regulation of viral genome replication and transcription [31]. It encodes 16 non-structural proteins, required for virus replication and pathogenesis, four structural proteins, including envelope (E), membrane (M), nucleocapsid (N), and spike (S) glycoprotein, and nine accessory factors that are thought to be involved in host response modulation [32,33,34] (Figure 2).

SARS-CoV-2 enters target cells by endocytosis mediated by interaction of viral S glycoprotein with human angiotensin-converting enzyme 2 (ACE2) that serves as host receptor [35]. This process requires cleavage of the S protein into two functional subunits (S1 and S2) by the host proteases, mostly transmembrane serine protease 2 (TMPRSS2) and cathepsin-L [2]. Functional S1 can bind the ACE2 receptor through its receptor biding domain (RBD), while S2 mediates viral fusion with the host cell membrane and release into the cytoplasm [35]. Besides ACE2, cellular glycans, integrins, neuropilin 1, and AXL have been involved as entry co-factors [36,37,38]. After cell entry, the viral genome is released into the cytoplasm, where it is directly translated by ribosomes into two large polyproteins, polyprotein (pp) 1a and pp1ab, which are cleaved by host and viral proteases to release nonstructural viral proteins, including viral RNA-dependent RNA polymerase, two viral proteases and other components of the viral replication, and transcription complex. At variance, structural proteins and accessory factors are translated via subgenomic RNA molecules, which are generated by template switch and discontinuous transcription of viral genome [34]. Viral genome replication requires synthesis of negative-sense copies of the full genome, which function as template for the generation of new positive-sense genomic RNA molecules [34]. Replication occurs in replication organelles, i.e., double membrane vesicles generated by subversion of host cell endomembranes [39,40]. The newly synthetized genomes are translated to generate viral proteins or packaged into new virions, which are released from infected cells through the lysosomal trafficking pathway [41]. Coronavirus proteins interact with a wide range of host proteins that are required for viral RNA synthesis, translation, and virus assembly, as well as with host factors involved in innate antiviral response, such as the interferon pathway, which is inhibited by SARS-CoV-2 ORF3b [42]. The function of SARS-CoV-2 nonstructural proteins is still largely unknown. However, it can be inferred from other coronavirus proteins, characterized by conserved enzymatic activities and functional domains across genera [43]. Some coronavirus nonstructural proteins have been involved in the modulation of host innate immune response, such as SARS-CoV-1 nsp1, which promotes cellular mRNA degradation and blocks host cell translation, resulting in innate immune response blockage [44,45], and SARS-CoV-1 nsp16 2′-O-methyl transferase, which shields viral RNA from recognition by host sensor molecules [46]. 

Productive viral replication leading to cytopathic effect (CPE) and cell death occurs in fully permissive target cells in the respiratory tract, while abortive infection associated with different mechanisms of cell and tissue injury might occur in extra-pulmonary sites. In this context, in vitro models of differentiated human cells, tissues, and organs represent excellent tools to investigate viral tropism and cell-specific permissiveness to SARS-CoV-2 infection and replication.

## 3. Pathogenesis of COVID-19 and the Needs of Model Systems

**Figure 3:**
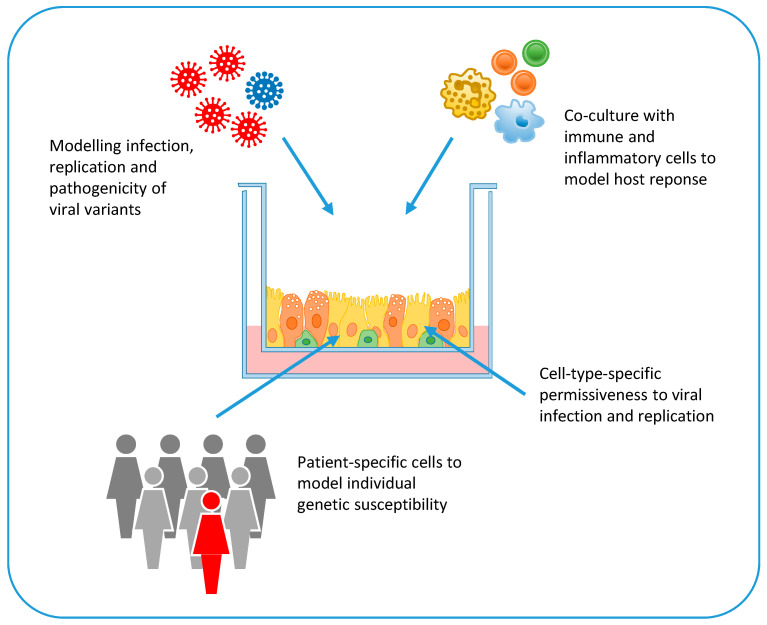
Applications of stem cell technology and organoids for in vitro modelling of SARS-CoV-2 infection and disease: Human cells grown as monolayers or organoids, which recapitulate the different cell types in tissues and organs, can be used to investigate cell-type-specific permissiveness to SARS-CoV-2 infection and replication. Cells and organoids can be grown in co-culture with immune cells to investigate the role of host immune and inflammatory response in disease pathogenesis. By using different SARS-CoV-2 genome variants, in vitro systems can be used to compare viral infection and replication efficiency and cytopathic effects. Finally, in vitro cell systems can be generated from patient-specific cells or genetically-modified cells to model individual genetic susceptibility to severe disease.

Pathogenesis of COVID-19 is largely unknown [47]. While, in most cases, SARS-CoV-2 infection triggers effective innate and adaptive immune responses, in some cases it induces a defective interferon (IFN) response and a pathological hyper-inflammatory condition that can lead to severe illness, septic shock, and multi-organ failure [47]. The hyper-inflammatory response is characterized by a cytokine storm, profound lymphopenia, and mononuclear cell infiltration in multiple organs, such as the lungs, heart, spleen, and kidneys [48,49]. Older people and people with co-morbidities have an increased risk to develop severe disease probably because of defective innate immune response and T cell activation. Bronchoalveolar lavage fluids of patients with severe disease present elevated levels of inflammatory cytokines and type I and III IFNs [50]. However, the IFN response is dysregulated and delayed in COVID-19 patients, probably because coronaviruses can evade recognition by host pattern recognition receptors and directly antagonize IFN signaling [51]. In the mouse model, sustained IFN type III production by lung dendritic cells upon viral recognition disrupts lung barrier function and predispose to lethal secondary bacterial infections [50]. The primary causes of COVID-19 associated mortality are cardiorespiratory failure and coagulopathy [52,53]. Postmortem examination of the lungs showed diffuse alveolar damage, endothelial injury, widespread thrombosis, and increased vascular angiogenesis [54]. In addition, brain involvement with pan-encephalitis and brainstem neural damage has been described [55,56]. Viral genome sequences have been detected in several organs and tissues of deceased COVID-19 patients, including the lungs, pharynx, heart, kidneys, gut, and brain [55,57]. This broad distribution reflects the distribution of the ACE2 receptor, which is expressed not only in alveolar epithelial type II cells [58], but also in the heart, kidneys, blood vessels, colon, and intestine [59]. Given the expression of ACE2 in a variety of tissues, human cell-based platforms are helpful to verify whether this receptor and other host entry and restriction factors are present in SARS-CoV-2 target cells, to evaluate viral tropism, to study the cell types permissive to SARS CoV-2 infection, and to model COVID-19 pathogenesis across multiple organ systems (Figure 3). In addition, the co-culture of these SARS-CoV-2 infection models with inflammatory and immune cells (e.g., macrophages, neutrophils, natural killer cells, lymphocytes) could provide valuable information on the role of local and systemic inflammatory responses to COVID-19 pathogenesis (Figure 3).

Both T and B cell responses are recognized as important for SARS-CoV-2 clearance. SARS-CoV-2-specific CD8^+^ T cells play a key role in killing infected cells, while CD4^+^ T cells produce IFN type II and cytokines that are required for recruitment of immune cells. The relevance of T cell response in the resolution of infection is highlighted by lymphopenia and impaired T cell responses occurring in patients with severe COVID-19 that result from delayed IFN response [60]. SARS-CoV-2 infection induces the production of neutralizing antibodies that protect against reinfection [48,61]. However, a subset of infected individuals, especially those who are asymptomatic or have mild symptoms, develop low level or no antibody response to SARS-CoV-2 [62]. 

The presence of genetic variants in SARS-CoV-2 genome has been investigated for the possible association with increased transmissibility and virulence. Coronavirus genome, including SARS-CoV-2 genome, is remarkably stable because of the 3′–5′ exonuclease and proofreading activity of nsp14 [63]. Nonetheless, several genome variants have emerged during the pandemic, some of which have been associated with increased fitness and transmissibility. For example, the D614G mutation in the spike glycoprotein of SARS-CoV-2 emerged in late February 2020 from southern Europe and rapidly spread and become the most prevalent genotype worldwide [64]. This variant, which was predicted to have a higher affinity to the human ACE2 receptor, was associated with higher viral load in the upper respiratory tract of patients, but not with increased disease severity [64], as well as with higher replication efficiency in the upper respiratory tract of infected hamsters, without causing more severe disease [65]. Another SARS-CoV-2 genetic variant carrying the N501Y mutation in the RBD domain and deletion of amino acids 69 and 70 in the S protein emerged in September 2020 in South East England and rapidly increased its prevalence in England, where it represented over 98% of sequences in December 2020 [66]. This variant was characterized by increased transmissibility, but no statistically significant differences in hospitalization, 28-day case fatality, and re-infection rates compared with the wild-type virus [66,67]. In vitro models of human (and non-human) cells and organs could be very useful to investigate and compare the tropism and fitness of SARS-CoV-2 variants (e.g., by monitoring the grow kinetics of a new genetic variants in competitive infection experiments with the wild-type virus; by investigating the pathogenic effects of a mutant viral protein in relevant host cells; by analyzing the infection and replication efficiency of a new viral variant in relevant target cells generated from different hosts) (Figure 3).

Severe life-threatening COVID-19, which strikes less than 1 in 1000 infected young individuals without comorbidities, is also the result of host genetic predisposition [68]. A genome wide association study involving 1980 patients with COVID-19 and respiratory failure from Italy and Spain identified significant associations with the AB0 blood group locus and a cluster of six genes at locus 3p21.31 encoding chemokine receptors and the sodium-imino acid transporter 1, which functionally interacts with ACE2 [69]. Another study based on whole genome sequencing or exome sequencing of 659 patients with life-threatening COVID-19 pneumonia, relative to 534 subjects with asymptomatic or mild infection, investigated the presence of genetic variants in 13 loci involved in innate immune responses and known to be mutated in patients with severe viral diseases [70]. Interestingly, this study identified an enrichment of loss-of-function variants in TLR-3- and IRF7-dependent type I IFN immunity in 3.5% of severe COVID-19 cases who had no history of prior severe infections [70]. A role of genetic defects of innate immunity in severe COVID-19 was also identified by whole exome sequencing in two brother pairs from two unrelated families who were admitted to ICUs for COVID-19 [71]. The four men carried rare loss-of-function variants in the *TLR7* gene, which impaired downstream type I and II IFN signaling [71]. The key role of host innate immune response in COVID-19 pathogenesis was further highlighted by the detection of neutralizing auto-antibodies against type I IFN in 135 of 987 (13.7%) patients hospitalized with severe COVID-19 pneumonia but not in patients with asymptomatic or mild disease and rarely in healthy pre-COVID-19 controls [72]. Notably, anti-IFN-antibodies neutralized the ability of IFN-α2 to block SARS-CoV-2 infection in vitro and were associated with low levels of type I IFN transcripts in vivo [72]. Stem cell technology and organoids represent crucial tools to verify in vitro the genetic basis of infectious disease susceptibility. For example, cells and tissue biopsies can be obtained ex vivo from individuals with specific genetic mutations or severe disease phenotype and from healthy control subjects. These cells are then grown or reprogrammed in vitro to generate the cells that are targeted in vivo by the pathogen, and used in infection experiments (Figure 3). Alternatively, mutations associated with susceptibility (or resistance) to severe infectious diseases can be investigated in vitro by exploiting gene editing tools (e.g., CRISPR/Cas9). In this case, specific mutations are introduced in pluripotent stem cells; then, the genetically modified cells are differentiated into the desired cell lineage or organoid for infection experiments (Figure 3).

## 4. Stem Cell Technology and Organoids for Infectious Disease Modelling

Induced pluripotent stem cells (iPSCs) are pluripotent cells reprogrammed from adult somatic cells into an embryonic-like state [73]. Being pluripotent, iPSCs just as embryonic stem cells (ESCs), can give rise to virtually any mature cell type belonging to the three germ layers ectoderm, mesoderm, and endoderm. Differently from ESCs, iPSCs can be obtained from a variety of cell sources and with different genetic backgrounds. Soon after their discovery, scientists from the virology field appreciated the potential of using these tools to model viral infections and to study viral pathogenesis in unprecedented ways. In fact, hPSCs, once differentiated into a specific mature phenotype, represent a more relevant human model, recapitulating the in vivo situation, compared to traditional tumorigenic or transformed cell lines. Moreover, human iPSCs (hiPSC)-based systems overcome the limitation of species-specificity of several viruses, which can hamper the interpretation of findings translated from animals to humans. Indeed, hiPSCs have been applied to model numerous infectious diseases [74]. For instance, soon after the WHO declared Zika virus (ZIKV) outbreak in Brazil a public health emergency of international concern on 1 February 2016, efficient ZIKV infection of hiPSC-derived human neural stem cell was demonstrated in vitro [75], supporting the possible link between ZIKV infection and microcephaly. Subsequently, hiPSC-derived neural progenitor cells (NPCs) have been widely used to investigate the mechanisms of ZIKV neuro-pathogenesis, associated microcephaly or to perform drug screenings [76,77,78,79,80]. 

Studies of viral interaction with human iPSC-derived cells have laid the foundation for recent studies using organoids. Organoids are in vitro 3D multicellular structures that mimic the corresponding in vivo organ, recapitulating its main features from a morphological and functional point of view. They consist of self-organized cell types with restricted lineage commitment that generate cell assemblies with architectural and functional characteristics of the related tissue. Organoids can be established form pluripotent stem cells, i.e., iPSCs and embryonic stem cells, or from adult stem cells derived from surgical specimens [81,82]. So far, several “organ-like” 3D systems have been developed from hPSCs: brain, optic cup/retina, salivary gland, thyroid, mammary gland, liver, pancreas, stomach, intestine, fallopian tube, endometrium, kidney, lung, blood vessel, some of which have been also derived from adult stem cells (for a comprehensive review, see ref. [83]). Relevant to SARS-CoV-2 infection, lung, intestinal and brain organoids have been very useful so far to provide additional evidence on virus-induced organ injury. Intestinal and brain organoids have been the first to be developed and benefit from well-established and highly reproducible protocols for their generation [84,85]. For intestinal organoids, after endoderm induction, mid- and hindgut tissues develop through the formation of spheroids that bud from the epithelium. These are further cultured giving rise to intestinal tissue with fully differentiated epithelial cells comprising all of the major intestinal cell types and intestinal mesenchyme. The cells are polarized, and produce and secrete mucus onto the apical surface [84]. Mini-brains are derived from hPSCs by allowing the formation of floating embryoid bodies and forcing only the neuroectoderm to develop. After embedding in a matrix, the tissues are cultivated in a spinning bioreactor that allows extensive growth and further maturation. Mature brain organoids are composed by neuroepithelial tissues with regions containing NPCs and neurons and exhibit large fluid-filled cavities reminiscent of ventricles [86]. For lung organoids, hPSCs are directed to endoderm differentiation and then to anterior foregut endoderm. Foregut spheroids self-aggregate and are cultured in a matrix, where they are directed to become airway-like organoids, containing cell types and structures that resemble the bronchi/bronchioles surrounded by lung mesenchyme and cells expressing alveolar-cell markers [87].

The organoid technology has been applied to study virus–host interactions. For example, by analyzing the interplay between epithelial cells and immune cells and pathogens, organoid-immune cell co-culture systems have been used to demonstrate the immunomodulatory properties of factors produced by epithelial cells [88,89], the role of immune cells and cytokines in maintaining tissue homeostasis [90], and the interactions between pathogen-activated immune cells and epithelial cells [91]. Organoids derived from hPSCs have been used to model infection of epithelial tissues. hPSC-derived intestinal and lung organoids, for instance, have been used to model, respectively, rotavirus and respiratory syncytial virus pathobiology [92,93]. iPSC-derived brain organoids, the so-called mini-brains, were very useful to reveal the pathogenesis mechanisms of ZIKV infection: it has been demonstrated that, in fact, the virus can impair growth and cause a disruption of cortical layers by replicating and inducing apoptosis in NPCs [94,95]. It is important to note that, albeit organoids represent a relevant organ model that overcome the limitations of species-specificity of viruses and provide faster and more robust outcomes, there are some limitations in the use of this technology. First, there are differences in the organoids compared to the corresponding organs as they are less complex and do not interact with the local environment of the body (immune cells, vasculature, nerves) as it physiologically happens in vivo for organs. Co-culture systems with other cell types might help to fill this gap, but these type of models are still in their infancy and need to be solidly established. Moreover, the observed diversity of the generated organoids, due to differences in protocols adopted for their generation or in the starting cell type, is still a relevant constrain. Nevertheless, organoids have a great potential and might help revolutionize virus–host interaction studies.

## 5. Screening for SARS-CoV-2 Tropism for Human Tissues and Cells

Human cells and tissues have been used to isolate SARS-CoV-2 in culture, to identify the cells targeted by infection, and to model COVID-19 pathophysiology. Notably, the first isolate of SARS-CoV-2 was obtained by inoculation of bronchoalveolar-lavage fluid samples on primary human airway epithelial cells [1]. These cells were cultured in vitro to generate well-differentiated, polarized cultures resembling in vivo pseudostratified mucociliary epithelium [1].

To explore systematically SARS-CoV-2 tropism and cell response to infection, Yang and colleagues derived multiple cell types and organoids from hPSCs, including hiPSCs and embryonic stem cells [96]. Specifically, they differentiated hPSCs into eight different cells types and organoids, i.e., pancreatic endocrine cells, liver organoids, endothelial cells, cardiomyocytes, macrophages, microglia, cortical neurons, and dopaminergic neurons. Experiments showed ACE2 expression in pancreatic α and β cells, hepatocytes, endothelial cells, cardiomyocytes, microglia, macrophages, and dopaminergic neurons, while cortical neurons expressed low levels of ACE2. Accordingly, efficient infection with a SARS-CoV-2 pseudo-typed vesicular stomatitis virus and with a SARS-CoV-2 isolate was demonstrated in pancreatic endocrine cells, liver organoids, cardiomyocytes, and dopaminergic neurons, while no or low virus entry was demonstrated in the other cell types [96]. SARS-CoV-2 infection of pancreatic endocrine cells upregulated chemokine expression and host genes involved in viral infection, while inducing cell apoptosis. However, the ability of SARS-CoV-2 to infect the endocrine pancreas in humans needs further investigation, since a deeper analysis of human pancreatic tissues did not detect ACE2 and TMPRSS2 proteins in α or β cells [97]. At variance, ACE2 protein was detected in the microvasculature of islet and exocrine tissue and in a subset of pancreatic ducts, whereas TMPRSS2 expression was restricted to ductal cells [97]. The ability of SARS-CoV-2 to infect the kidney has been poorly characterized. Experiments showed SARS-CoV-2 infection of kidney organoids, which was inhibited by treatment with human recombinant soluble ACE2 [98]. 

**Figure 4:**
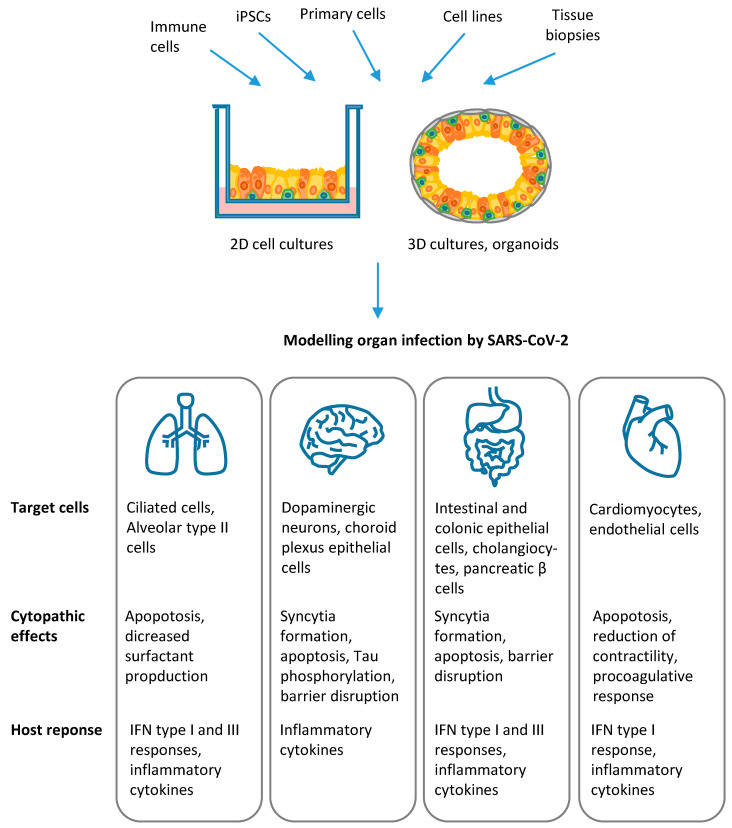
Modelling organ infection by SARS-CoV-2: SARS-CoV-2 infection of human lung epithelia, central nervous system, gastrointestinal tract, and cardiovascular system has been investigated by using stem cell technology and organoids. The architecture and physiology of different tissues and organs can be recapitulated in human 2D and 3D cultures of differentiated cells and organoids. These in vitro cell systems are generated from induced pluripotent stem cells (iPSCs), primary cells, cell lines and ex vivo tissue biopsies. Co-culture with immune cells allows to investigate the complex interplay with host inflammatory and immune response. The results of studies published in the literature, which demonstrated the potential of these in vitro models to dissect viral tropism and host cell response, are summarized in the figure.

Infection of human lung epithelia, central nervous system, heart, vasculature, and gastrointestinal tract has been investigated by several studies, which demonstrated the great usefulness of stem cell technology and organoids to dissect viral tropism, the mechanisms of infection and host cell response, as detailed in the following paragraphs and summarized in Figure 4.

## 6. Modelling SARS-CoV-2 Infection in the Lung

A variety of epithelial cells of the respiratory tract ranging from the nasal sinuses to the lung alveolar epithelial cells express ACE2 and its associated protease TMPRSS2 and are permissive to SARS-CoV-2 infection [99,100]. Expression of ACE2 is particularly high in the nasal cavity and decreases progressively from the upper respiratory tract to the alveoli [99,100]. In the lungs, ACE2 and TMPRSS2 are expressed mainly in ciliated cells in bronchial tract and, at lower levels, in alveolar type II cells, but not in differentiated lung alveolar type I cells [59,99,101], in agreement with the higher efficiency of SARS-CoV-2 infection and replication in the upper respiratory tract than in the distal lung [59,99]. 

Thus, proximal airway epithelial cells represent the first site of viral infection and replication, as shown by efficient infection of ciliated cells in organoid-derived bronchial airway cultures [102]. However, in patients with COVID-19 pneumonia, ARDS results from the damage of the distal lung epithelium, and, in particular, of alveolar epithelial type II cells, which represent the progenitor cell population of the lung [99]. It is conceivable that infection of ciliated cells triggers inflammatory and interferon responses, leading to upregulation of ACE2 locally in alveolar cells [103]. 

The permissiveness of human lung alveolar epithelial cells to SARS-CoV-2 and their response to viral infection was investigated in different in vitro models based on human primary cells cultures or on the differentiation of pluripotent stem cells. In the first approach, alveolar organoids were generated from primary small airway basal cells [102], from single adult human alveolar epithelial type II or KRT5+ basal cells [101,104], or from multipotent SOX2+SOX9+ lung bud tip progenitor cells, which could be differentiated into both alveolar-like and bronchiolar-like cells [105]. In the second approach, lung organoids were obtained by a stepwise strategy, including the progressive differentiation of pluripotent embryonic stem cells into definitive endoderm, followed by specification to anterior foregut endoderm, lung progenitor cells, and finally lung organoids [106]. Human alveolar epithelial type II cells were also obtained by directly differentiating from either human embryonic stem cells or iPSCs and selected for expression of the differentiation marker surfactant protein-C [100]. In these models, alveolar epithelial type II-like cells were grown in 3D Matrigel cultures as monolayered epithelial spheres or as 2D air–liquid interface cultures, characterized by apical-basal polarization and barrier integrity [100,101,105,106], or as organoids in long-term feeder-free, chemically defined culture systems [104]. These cells had *ACE2* and *TMPRSS2* expression levels similar to the adult stage, with *TMPRSS2* expression more widespread and robust than that of *ACE2* [100,101,104,105,106]. Experiments showed that SARS-CoV-2 could infect and replicate in alveolar epithelial type II cells grown as either 3D organoids, 3D spheres, or 2D air–liquid interface cultures in which viral infection and release of infectious virus occurred predominantly from the apical side, where ACE2 protein is located [100,101,104,105,106]. Productive SARS-CoV-2 infection was observed also in organoids generated from distal-lung basal cells, representing lung progenitor cells [104,105]. SARS-CoV-2 infection induced cytopathic effects with apoptosis both in infected and neighboring cells and a robust induction of host antiviral response genes, like IFN type I and type III, the key transcription factors STAT1 and STAT2 that regulate downstream signaling pathways, IFN receptors and other interferon-stimulated genes (ISGs) attributed to type I and type III IFN responses, NF-κB-mediated inflammatory signaling, and chemokine signaling pathway [100,101,105,106]. At variance, specific functions of alveolar epithelial type II cells, such as surfactant gene expression, as well as genes involved in DNA replication and cell cycle were downregulated in infected cells, while apoptosis-related genes were upregulated [101]. However, while primary cell cultures exhibited robust IFN response [101,105], alveolar epithelial type II cell organoids and 2D air–liquid interface cultures derived from pluripotent stem cells had a moderate response [100,106]. 

Alveolar organoids and proximal airway air liquid interface cell culture systems are useful to test antiviral compounds against SARS-CoV-2 or the effect of factors that increase the risk of lung damage leading to severe COVID-19. The following drug candidates for the treatment of COVID-19 have been tested so far: IFN type I, IFN type III, remdesivir, camostat mesylate (a TMPRSS2 inhibitor), E-64d (an inhibitor of the endosomal cysteine proteases cathepsin B and L), a library of FDA-approved drugs (the Prestwick collection) [100,105,106]. Both remdesivir and camostat mesylate displayed antiviral effect and reduced SARS-CoV-2 N levels [100]. Among FDA-approved drugs, imatinib, mycophenolic acid, and quinacrine dihydrochloride decreased SARS-CoV-2 infection of hPSC-derived lung organoids, probably by inhibiting SARS-CoV-2 entry [106]. Pre-treatment with IFN-λ1 abrogated viral replication in bronchioalveolar organoids, while treatment at 24 h post infection reduced infectious virus by ~5 logs [105]. Likewise, pretreatment with IFN-α and IFN-γ significantly reduced viral titers of alveolospheres [101]. 

Among factors that increase the risk of COVID-19, the effects of cigarette smoke and androgens have been investigated by using in vitro models of SARS-CoV-2 infection of the lungs [107,108]. Cigarette smoke is the most important cause of chronic lung disease and is associated with an increased risk of severe COVID-19 [109]. In air–liquid interface cultures derived from primary human nonsmoker airway basal stem cells, exposure to cigarette smoke before SARS-CoV-2 infection leads to 2- to 3-fold increase in viral load, increases the number of infected and apoptotic cells, prevents the normal airway basal stem cell repair response, and blunts IFN response [107]. Androgen signaling is a key regulator of ACE2 expression and male gender in adults is a risk factor of adverse COVID-19 outcome. In vitro studies confirmed the association between androgens and severe COVID-19, since treatment with the antiandrogenic drugs finasteride, ketoconazole, and dutasteride reduced ACE2 expression and protected human embryonic stem cell-derived lung organoids against SARS-CoV-2 infection [108].

## 7. Modelling SARS-CoV-2 Infection in the Central Nervous System

Neurologic symptoms, including headache, altered mental status, neuropsychiatric disorders, ageusia, and anosmia, are frequent in patients with COVID-19 [109,110]. Neuropathological examination of autopsies of patients with SARS-CoV-2 infection showed hypoxic injury in the brain with loss of neurons, but no encephalitis or other specific damage consistent with viral infection [55]. Moreover, despite some reports of detection of SARS-CoV-2 in the brain and cerebrospinal fluid of patients with COVID-19 [55,57,111], it is still unclear whether the virus can infect the central nervous system. In particular, it still remains to be elucidated whether these symptoms are a direct consequence of the replication of the virus in neural cells, are due to post-infectious immune-mediated disease, or are the result of systemic disease [112,113]. Conflicting preliminary data have been reported on SARS-CoV-2 neurotropism in in vitro systems derived from hPSCs, including data on expression of the entry receptor ACE2 in neural stem cells/NPCs and in mature neurons [96,114,115,116,117,118,119,120]. ACE2 expression has been also reported in 3D brain spheres and in brain organoids [117,118], whereas other researchers detected the presence of the receptor confined in choroid plexus (ChP) epithelial cells of brain organoids, but not in neurons and NPCs [119,120]. Although these conflicting data may be ascribable to the different sensitivity of the methods employed, in line with this varying pattern of ACE2 expression, susceptibility and permissiveness of neural cells to SARS-CoV-2 was also investigated with conflicting results in 2D cultures of neural cells, i.e., NPCs, neurons, and astrocytes. Infection and replication of SARS-CoV-2, leading to reduced cell viability, has been reported in NPCs [115], while other researchers reported no infection or replication in the same cell types [118,119]. 2D neurons cultures have been shown to be resistant to SARS-CoV-2, with only a sparse infection of neurons and astrocytes observed in few cases and only by drastically increasing the multiplicity of infection [96,119,120]. Alternatively, genetically engineered ApoE4/4 hiPSCs-derived astrocytes and neurons displayed an increased rate of SARS-CoV-2 infection with astrocytes exhibiting enlarged size and elevated nuclear fragmentation [121]. On the opposite, other groups observed infection, but not replication, in these neuronal cultures [117,118]. These inconsistent results in neural cell viral susceptibility may be the results of different adopted infection viral loads, differentiation protocols, and neural cell maturity. While brain spheres represent an earlier phase of brain development, 3D brain organoids represent a more advanced stage, displaying cells with a more mature phenotype. SARS-CoV-2 can infect and replicate in brain spheres, with an increase of viral RNAs, viral particles release, and the detection of viral particles in vacuoles by electron microscopy [114,115]. Similarly, SARS-CoV-2 can infect brain organoids and in particular mature neurons that are present in both peripheral and deeper regions, with syncytia formation and neuronal cell death [115]. Wang and colleagues [121] recently demonstrated that the viral nucleocapsid RNA level was substantially higher in SARS-CoV-2-infected brain organoids with astrocytes compared to brain organoids without astrocytes and in support to this result neurons co-cultured with astrocytes exhibited higher infection rate compared to neurons cultured alone, suggesting that astrocytes might have a role in boosting SARS-CoV-2 infection in neurons. Moreover, Ramani and colleagues [118] reported an aberrant phosphorylation of Tau in SARS-CoV-2-positive neurons of brain organoids, but inability of the virus to actively replicate, thus suggesting that an abortive cycle of the virus could be able to dysregulate neuronal function anyhow. In contrast with these previous works, two groups demonstrated concomitantly the inability of SARS-CoV-2 to infect and replicate in cerebral or region-specific brain organoids (cortical, hippocampal, hypothalamic, and midbrain organoids) indicating ChP epithelial cells as the target cells for the virus [119,120]. These cells are responsible for the production of CSF and their infection might represent an entryway of the virus to the central nervous system. By developing ChP specific brain organoids, they both demonstrated the ability of SARS-CoV-2 to infect and replicate at high rates almost exclusively in these cells types [119,120]. Infection leads to the formation of syncytia and to a significant increase in cell death, both in infected and uninfected cells, with an impaired physiological function of the epithelium causing tight junction disruption, transcriptional dysregulation of inflammatory cellular responses, and compromised blood–CSF barrier function [119,120]. Overall, these studies demonstrate that SARS-COV-2 can infect neural cells and brain organoids but the ability of the virus to replicate in these in vitro systems and to cause neurologic disease in vivo remains to be further elucidated.

## 8. Modelling SARS-CoV-2 Infection in the Cardiovascular System

Cardiovascular and thrombotic complications are major causes of morbidity and mortality in patients with severe COVID-19 [53,54]. Pathological examination of tissues from deceased COVID-19 patients suggests SARS-CoV-2 infection of myocardial and endothelial cells [122], but it is conceivable that indirect effects of systemic inflammatory and immunological hyper-responses play a critical role in disease exacerbation [123,124]. The direct effect of viral infection in cardiac cells was demonstrated in vitro in human iPSC-derived cardiomyocytes grown as monolayers [125]. SARS-CoV-2 can infect and replicate in these cells via ACE2, leading to a reduction of functional contractility and induction of cytokine and chemokine expression and apoptotic cell death [126]. 

At variance with occasional findings in lung microvascular endothelial cells of COVID-19 patients [54], in vitro grown endothelial cells do not express ACE2 [126] and are poorly susceptible to SARS-CoV-2 infection [127]. However, upon ACE2 overexpression, primary endothelial cells are productively infected by SARS-CoV-2, which triggers procoagulative and inflammatory responses [127]. Consistently, COVID-19 patients do not develop hemorrhagic disease that would result from lytic viral infection and massive disruption of the endothelium. On the other hand, exposure of primary lung microvascular endothelial cells to plasma samples obtained from patients with severe COVID-19 causes loss of barrier function, suggesting that endogenous plasma factors rather than direct injury by viral infection are probably determinant in disease progression [128].

## 9. Modelling SARS-CoV-2 Infection in the Gastrointestinal System

Gastrointestinal symptoms and fecal shedding of SARS-CoV-2 RNA are common in COVID-19 patients and are the consequence of viral infection of the gut epithelium. In fact, pathological analysis of human tissues showed the presence of SARS-CoV-2 in intestinal and colonic epithelial cells [106], both characterized by high levels of ACE2 and TMPRSS2 expression [129]. 

To investigate the permissiveness of human intestinal and colonic epithelial cells to SARS-CoV-2, researchers generated human small intestinal and colonic organoids from primary gut stem cells or tissues [102,130] and pluripotent stem cells [106], respectively. Experiments with human small intestinal organoids showed that SARS-CoV-2 productively infects mature enterocytes and leads to apoptotic cells death, while enteroendocrine and goblet cells are unaffected [102,130]. Infection occurs from the apical surface, where the ACE2 receptor is highly expressed [130]. Infection triggers membrane cell fusion and syncytia formation mediated by cleavage of the S protein Qmediated by TMPRSS2 and TMPRSS4 serine proteases, both highly expressed in human intestinal enterocytes [130]. Then, the virus is released in the lumen at the basolateral and apical sites of cells [102,130]. In enterocytes, SARS-CoV-2 infection induces the expression of cytokines and ISGs related to IFN type I and III responses, such as IP-10/CXCL10 and ISG15 [102,131]. SARS-CoV-1 showed similar infection and replication efficiency of SARS-CoV-2 in intestinal enterocytes and induced similar cytopathic effects, while it did not induce IFN type I and III responses [102]. Furthermore, in human colonic organoids, enterocytes express the highest levels of ACE2 and TMPRSS2 and are the cell types most susceptible to SARS-CoV-2 infection and cytopathic effects [106]. Notably, SARS-CoV-2 can also infect and replicate in small intestinal organoids established using crypts isolated from the intestines of *Rhinolophus sinicus* bats, the natural host for SARS-CoV-like coronavirus [131]. 

Liver damage is another common condition in patients with severe COVID-19 and could be the consequence of direct viral infection of the liver or indirect effects of systemic inflammatory response [49]. In vitro, SARS-CoV-2 infection of hepatocyte and cholangiocyte organoids upregulates inflammatory pathways and chemokine expression, consistently with findings in COVID-19 autopsy samples [96] and with evidence of liver damage in COVID-19 patients. In the healthy liver, ACE2 expression is particularly high in cholangiocytes, thus representing a potential target of SARS-CoV-2 infection and direct injury. SARS-CoV-2 infection of human cholangiocytes was demonstrated in an in vitro model of liver ductal organoids, derived from liver bile duct-derived progenitor cells grown in a 3D culture system [132]. Infected cells overexpressed inflammatory and chemokine genes, formed syncytia and underwent apoptotic cell death with disruption of the barrier and bile acid transporting functions of bile ductal epithelium [132].

## 10. Conclusions

Stem cell technology and organoids have provided a valuable contribution to the understanding of SARS-CoV-2 cell tropisms and the mechanism of disease in the human host, supporting and clarifying findings from clinical studies in infected individuals [27]. In vitro models of lung epithelia showed that alveolar epithelial type II cells, but not differentiated lung alveolar epithelial type I cells, are key targets of SARS-CoV-2 infection, which induces cell apoptosis and triggers inflammatory and innate antiviral responses, while impairing specific cell functions, like surfactant production [100,101,102,103,104,105,106]. Experiments with human small intestinal organoids and colonic organoids showed that the gastrointestinal tract is another relevant target for SARS-CoV-2. The virus can infect and replicate in enterocytes in the small intestine and colon and cholangiocytes, inducing cell damage and inflammation [96,102,106,130,132]. Direct viral damage was also demonstrated in in vitro models of human cardiomyocytes [125,126], astrocytes, and ChP epithelial cells [119,120]. At variance, endothelial cells and neurons are poorly susceptible to viral infection [118,119,127], thus supporting the hypothesis that neurological symptoms and vascular damage result from the indirect effects of systemic inflammatory and immunological hyper-responses to SARS-CoV-2 infection [128].

## Data Availability

Not applicable.
